# Change in scar visibility in LGE-MRI with time after RF ablation procedure

**DOI:** 10.1186/1532-429X-17-S1-P233

**Published:** 2015-02-03

**Authors:** Euguene Kholmovski, Sathya Vijayakumar, Alan K Morris, Ravi Ranjan, Nassir F Marrouche

**Affiliations:** Surgical Services Clinical Program, Intermountain Healthcare, Salt Lake City, UT USA; UCAIR, Department of Radilogy, University of Utah, Salt Lake City, UT USA; CARMA Center, University of Utah, Salt Lake City, UT USA

## Background

Atrial fibrillation (AF) is the most common cardiac arrhythmia affecting more than 2 million people in North America. Radio-frequency (RF) and cryo ablations of the left atrium (LA) are effective for symptomatic, drug refractory AF patients. Reported success rates of the procedure vary significantly with AF recurrences ranging from 25-50%. Many AF recurrences occur early after the procedure. However, considerable fraction of recurrence happens later (> 3 months after the procedure) when post-ablation scar is believed to be stable.

Late Gadolinium Enhancement (LGE) MRI is used to assess post-ablation scar in AF patients treated using RF and cryo ablations. The main aim of this work was to study how the visibility of post-ablation scar in LGE-MRI changes with time after RF ablation procedure.

## Methods

50 patients who underwent a single RF ablation procedure were imaged by LGE-MRI to assess scar at successive time points of 3-6, 9-15 months, 18-27 months, and > 30 months post-procedure. LGE images of LA were acquired about 20 minutes after contrast injection (0.1 mmol/kg, Gd-BOPTA) using a 3D respiratory navigated, inversion recovery prepared GRE pulse sequence. Typical scan parameters were TR/TE=3.1/1.4 ms, flip angle of 14, voxel size=1.25x1.25x2.5 mm. Inversion pulse was applied every heart beat and fat saturation applied immediately before data acquisition limited to 15% of RR cycle.

Wall of left atrium (LA) was manually segmented and post-ablation scar was identified by analysis of signal intensity distribution of the segmented LA wall. Scar identification was visually validated and corrected if required by an experienced operator. The visibility of scar was quantitatively assessed using Contrast to Noise Ratio (CNR) and Image Intensity Ratio (IIR) metrics. CNR was computed as the difference between mean signal of scar and mean signal of blood in LA cavity divided by the standard deviation of the blood signal in LA cavity. IIR was calculated as a ratio of mean signal of scar and mean signal of LA blood.

## Results

**Figure 1:**
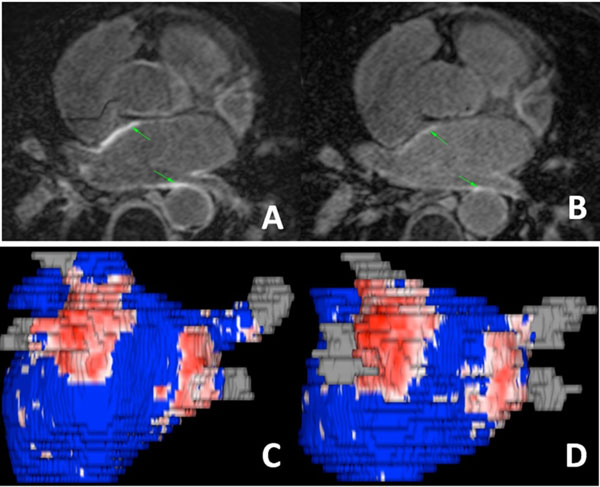
Scar detected by LGE-MRI performed 3 and 18 months post ablation.

**Figure 2:**
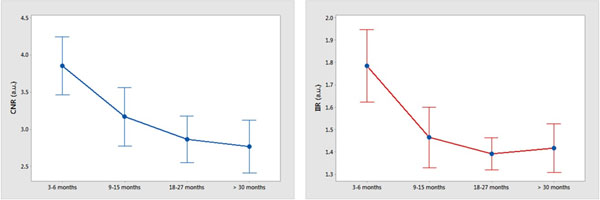
Dependence of post-ablation scar CNR and IIR in LGE-MRI on time after RF ablation. The mean values and 95% confidence intervals are shown.

Similar spatial distribution of post-ablation scar was observed in LGE-MRI studies acquired at different times post ablation (Figures 1). However, the visibility of scar in LGE-MRI significantly reduces with time post ablation (Figure 2). It was found that both CNR and IIR are significantly decreased (p< 0.01) between LGE MRI studies performed 3-6 months post-ablation and later.

## Conclusions

The visibility of scar in LGE-MRI significantly reduces with time post ablation. These results indicate that scar tissues undergo physiological changes affecting amount of extra-cellular space and contrast kinetics as late as 2 years post ablation. This scar remodeling may be responsible for late AF recurrences.

## Funding

This study was supported in part by Marrek Inc.

